# Prefrontal Cortex Involvement during Dual-Task Stair Climbing in Healthy Older Adults: An fNIRS Study

**DOI:** 10.3390/brainsci11010071

**Published:** 2021-01-07

**Authors:** Talia Salzman, Ahmed Aboualmagd, Hawazin Badawi, Diana Tobón-Vallejo, Hyejun Kim, Lama Dahroug, Fedwa Laamarti, Abdulmotaleb El Saddik, Sarah Fraser

**Affiliations:** 1 School of Human Kinetics, Faculty of Health Science, University of Ottawa, Ottawa, ON K1N 7K4, Canada; tsalz084@uottawa.ca (T.S.); akim009@uottawa.ca (H.K.); 2 Interdisciplinary School of Health Sciences, Faculty of Health Science, University of Ottawa, Ottawa, ON K1N 7K4, Canada; amahm036@uottawa.ca (A.A.); ldahr052@uottawa.ca (L.D.); 3 Multimedia Communications Research Laboratory (MCRLab), School of Electrical Engineering and Computer Science, University of Ottawa, Ottawa, ON K1N 7K4, Canada; hbada049@uottawa.ca (H.B.); flaam077@uottawa.ca (F.L.); elsaddik@uottawa.ca (A.E.S.); 4 Electronics and Telecommunications Engineering Department, Universidad de Medellín, Medellín 050030, Colombia; dtobon@udem.edu.co

**Keywords:** cognitive aging, cognitive function, executive function, functional near-infrared spectroscopy (fNIRS), gait, neuroimaging, older adult, prefrontal cortex (PFC), stair climbing

## Abstract

Executive function and motor control deficits adversely affect gait performance with age, but the neural correlates underlying this interaction during stair climbing remains unclear. Twenty older adults (72.7 ± 6.9 years) completed single tasks: standing and responding to a response time task (SC), ascending or descending stairs (SM_up_, SM_down_); and a dual-task: responding while ascending or descending stairs (DT_up_, DT_down_). Prefrontal hemodynamic response changes (∆HbO2, ∆HbR) were examined using functional near-infrared spectroscopy (fNIRS), gait speed was measured using in-shoe smart insoles, and vocal response time and accuracy were recorded. Findings revealed increased ∆HbO2 (*p* = 0.020) and slower response times (*p* < 0.001) during dual- versus single tasks. ∆HbR (*p* = 0.549), accuracy (*p* = 0.135) and gait speed (*p* = 0.475) were not significantly different between tasks or stair climbing conditions. ∆HbO2 and response time findings suggest that executive processes are less efficient during dual-tasks. These findings, in addition to gait speed and accuracy maintenance, may provide insights into the neural changes that precede performance declines. To capture the subtle differences between stair ascent and descent and extend our understanding of the neural correlates of stair climbing in older adults, future studies should examine more difficult cognitive tasks.

## 1. Introduction

Stairs have been identified as a common source of injurious falls amongst older adults [1]. From a neural perspective, stair ambulation may be increasingly challenging with advancing age due to structural and metabolic changes in the prefrontal cortex (PFC) [2,3]. As such, cognitive functions, such as executive functions, which are supervised by the PFC, may be less efficient at processing challenging cognitive demands. Studies that have examined the relationship between executive functions and gait in older adults have demonstrated that executive function deficits are associated with declines in gait performance [4,5,6,7]. Compared to overground walking, stair ambulation may be highly demanding because it involves elements of dynamic balance, lower body strength and attention [8,9]. Additionally, for both ascent and descent, motor planning begins prior to reaching the first step and continues thereafter to ensure precise foot placement and the proper integration of sensory and visual information [10,11,12]. Interestingly, older adults who report a fear of falling may increase their handrail usage to ensure stability on stairs [9,13]. Older adults equally suggest that stair ascent and descent pose separate challenges [12,13]. For example, greater balance compensation is required during stair descent, whereas ascent involves greater physical exertion to counteract gravitational forces [1,10]. These differences may also account for an increased incidence of falls during stair descent and slower gait speeds during stair ascent [12].

The dual-task paradigm may be used to better understand executive functioning during stair ambulation by examining an individual’s capacity to manage two tasks simultaneously [14,15]. When two overlapping processes compete for the same cognitive resources, performance on one or both tasks may suffer [16]. This is known as a dual-task cost which can be assessed using a variety of cognitive and motor performance measures. For example, older adults have demonstrated reduced vocal response times during a reaction time task while ambulating stairs [17]. Similar findings were obtained during a standing balance task, whereby decreasing the base of support and increasing instability led to slower response times in older adults [18]. Other studies have focused primarily on stair descent, where older adults decreased their gait speed compared to overground walking [19] and while responding to a mental arithmetic task [20]. Accuracy scores proved to be more dependent on cognitive task difficulty, such that higher scores were achieved during response time tasks [21] compared to serial subtractions [22] and working memory tasks [23] during overground walking. Thus, performance costs during dual-tasks may offer an indirect measure of executive functioning and allow for further insight into the neural mechanisms involved in stair ambulation.

In contrast, our knowledge of direct measures of neural activation on stairs has been limited by the restrictive nature of most neuroimaging techniques. However, functional near infra-red spectroscopy (fNIRS) has emerged as an important tool to measure neural activation because it is robust to motion artifacts and does not limit participant mobility [24,25]. fNIRS uses the principle of neurovascular coupling to measure the changes in cerebral oxygenation (∆HbO2) and deoxygenation (∆HbR) following a neural stimulus. When a series of vascular events are initiated to mitigate the increased metabolic demand of oxygen, the changes in cerebral blood flow and oxygen metabolism can be coupled and used as a neurophysiological marker to detect changes in brain activation [26]. PFC activity during overground walking and obstacle negotiation has been well documented in the literature, such that greater motor task complexity is associated with greater PFC activation [24,27,28]. Similarly, studies have demonstrated that older adults exhibit greater PFC activation during dual-task walking compared to walking alone [15,29,30]. This is in line with the revised scaffolding theory of aging and cognition (STAC-r) which suggests that older adults may adapt to age-related neurodegeneration by recruiting additional neural networks [31]. Therefore, stronger and bilateral recruitment of the PFC can be expected in older versus younger adults to maintain a high level of cognitive and motor performance during stair ambulation.

This study builds upon previous behavioral work that examined changes in gait speed and response time performance in older adults during stair negotiation [17,20]. The purpose of this study was to evaluate the hemodynamic response (∆HbO2 and ∆HbR) and performance (gait speed, response time and response accuracy) changes in older adults under single and dual-task stair ascent and descent. It was hypothesized that dual-tasks are more complex than single tasks and will invoke worse cognitive (e.g., response time and response accuracy) and motor (e.g., gait speed) performance due to a greater reliance on executive functions and the PFC. Similarly, stair ascent is more physically demanding, which may result in worse motor performance, whereas stair descent requires more planning and conscious attention, which may lead to worse cognitive performance. From a neural perspective, greater ∆HbO2 and ∆HbR are expected during the dual- compared to single tasks in line with the STAC-r neural compensation theory [31]. In addition, stair descent may require greater cognitive control than stair ascent, which is expected to elicit a greater hemodynamic response change in the PFC.

## 2. Materials and Methods

### 2.1. Participants

Twenty healthy older adults over the age of 60 (72.7 ± 6.9 years, 14 females) were recruited from community centres across Ottawa (Canada). Each participant was screened over the phone to determine their eligibility for the study. Participants that were deemed eligible also provided their age, gender, and years of education. The inclusion criteria were: (1) the ability to walk 15 m without assistance (e.g., cane); (2) no neuromuscular pain that could negatively affect stair ambulation; (3) right-handedness; and (4) no hearing aids or impairments that could affect one’s ability to respond to the cognitive task. This study was approved by the University of Ottawa Research Ethics Board (H-06-18-662) and was conducted in accordance with the Declaration of Helsinki. All participants provided written informed consent prior to participating.

### 2.2. Equipment

Neural measures were collected using an OctaMon fNIRS device (Octamon, Artinis, The Netherlands) and optodes were placed in reference to the modified International EEG 10–20 system to ensure the device was accurately placed on PFC [32]. The OctaMon uses continuous wave near-infrared spectroscopy to measure HbO2 and HbR light attenuation at 760 and 850 nm from eight emitters and two detectors.

Custom in-shoe smart insoles were designed and validated against the Tekscan Strideway system to measure gait [33,34]. Each insole was embedded with 12 force sensitive resistors (model FSR-402) that were spread across the heel (3), midfoot (1) and forefoot (8). The smart insoles were also standardized following the ISO/IEEE 11073 Personal Health Devices standards [35]. During the experiment, plantar pressure sensor readings were collected using a Bluetooth enabled smartphone application. To ensure consistent measurements across participants, small and large insoles were designed to accommodate different shoe sizes and were inserted into adjustable sandals.

**Figure 1:**
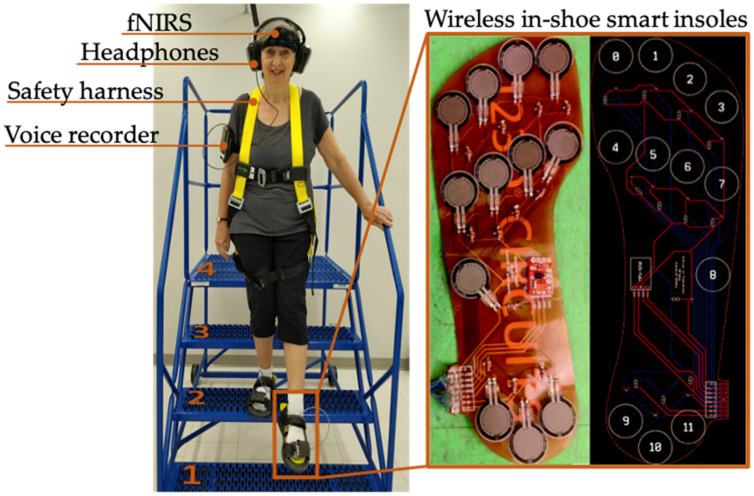
Example of a participant descending the stairs. Participants wore wireless in-shoe smart insoles with 12 force sensitive resistors dispersed across the forefoot, midfoot and heel (adapted from Badawi et al., 2019). The stairs were composed of four steps (25 cm riser, 30 cm tread, 91 cm width) and were located in a well-lit room. Participants also wore an fNIRS device, wireless headphones, a safety harness, and a voice recorder throughout the experiment.

The walking pathway was set up in a well-lit room and was composed of a 3.7 m walkway followed by a flight of four stairs. Handrails were located on either side of the stairs to support participants if they felt unstable. For the duration of the experiment, participants wore: (1) an fNIRS device, (2) a safety harness to protect them from vertical falls, (3) wireless headphones to hear the cognitive-auditory task, and (4) a voice recorder on their upper arm to record vocal response times and accuracy scores (Figure 1).

### 2.3. Experimental Design

**Figure 2:**
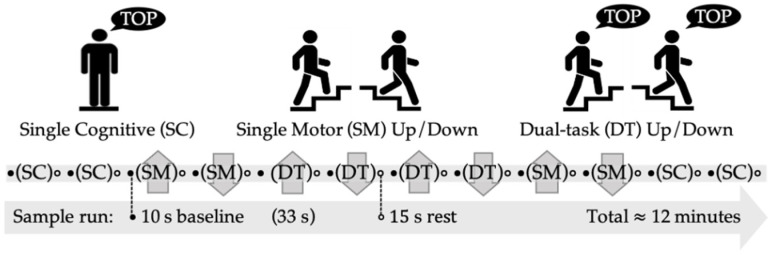
Sample run composed of 12 counterbalanced blocks. The single cognitive (SC) blocks had participants stand and perform a simple reaction time task. The single motor (SM) blocks had participants ascend or descend the stairs. The dual-task (DT) blocks had participants ascend or descend the stairs while responding to a simple reaction time task. Each block was preceded by a 10 s baseline and followed by a 15 s rest period.

A blocked design was used to measure the hemodynamic changes across three different tasks: single cognitive (SC), single motor (SM), and dual-task (DT) (Figure 2). The SM and DT conditions were further subdivided into two components, up and down, to differentiate stair ascent and descent. A run was made up of 12 counterbalanced blocks each lasting 33 s and preceded by a 10 s baseline. A 15 s rest period followed each block to allow the hemodynamic response sufficient time to return to the baseline before proceeding to the next block [36].

During the SC task, participants were asked to stand while responding to a simple reaction time task (SRT). The SRT task required participants to listen and respond to a random sequence of beeps by saying the word “top” as fast as possible following each stimulus [18,37]. During the SM ascent block (SM_up_), participants walked 3.7 m and then ascended a flight of four stairs. This was repeated in reverse for the SM descent block (SM_down_), whereby participants descended a flight of four stairs and then walked 3.7 m until a designated finish line. Similar to the SM task, the dual-task (DT) was divided into ascent (DT_up_) and descent (DT_down_) blocks, however, participants were asked to simultaneously respond to the SRT cognitive task. During the DT, participants were instructed to pay equal attention to stair ambulation and responding to the SRT stimuli [38]. After completing the dual-tasks, participants were asked to subjectively report out of 100% how much emphasis they placed on stair ambulation versus responding to the SRT task. In all conditions involving stair ambulation (SM and DT), participants were allowed to use the handrail as needed; this was based on previous stair climbing research with older adults [17].

### 2.4. Cognitive and Motor Test Battery

After completing the experiment, the experimenter administered standardized neuropsychological tests to assess baseline cognitive abilities that may play a role in dual-tasking. The test battery included: (1) the Montreal Cognitive Assessment (MoCA) [39], (2) Digit Forward and Backward [40], (3) the Digit Symbol Substitution Test [40], and (4) the Trail Making Test (TMT) part A and B [41]. The MoCA is a screening tool used to identify older adults who are at risk of cognitive impairment. It is scored out of 30, whereby scores ≥26 reflect healthy cognition. Digit Forward and Backward evaluates working memory, while the Digit Symbol Substitution Test assesses processing speed. In addition, TMT parts A and B are timed tests used to measure task switching and executive functioning, respectively. Furthermore, the Falls Efficacy Scale—International (FES-I) is a questionnaire that uses a 4-point Likert scale to evaluate questions on fear of falling in community dwelling older adults including on stairs [42]. Lastly, the Geriatric Depression Scale (GDS) is a questionnaire used to assess depression in older adults, which is known to affect the PFC [43]. In the case of the FES-I and the GDS, higher scores indicate a greater fear of falling and increased indications of depression (Table 1).

### 2.5. Data Processing

fNIRS data were sampled at 10 Hz in Oxysoft (v3.0.97.1, Artinis, The Netherlands) and was visually inspected to confirm the presence of synchronous waveforms (e.g., no abnormal spikes). Outliers exceeding 2.5 *SD* from the mean were removed and replaced by a zero value. The modified Beer–Lambert law was applied to the HbO2 and HbR raw intensities using a 6.61 differential pathlength factor (DPF) across all participants [44]. The derived concentrations were then preprocessed offline using a custom MATLAB (2018a) script in which ∆HbO2 and ∆HbR were measured from a baseline to the task-evoked hemodynamic response. In line with similar studies, physiological noise (i.e., heartbeat and breathing) and motion artifacts were filtered from the signal using a Butterworth bandpass filter set between 0.01–0.14 Hz [30,45,46].

**Figure 3:**
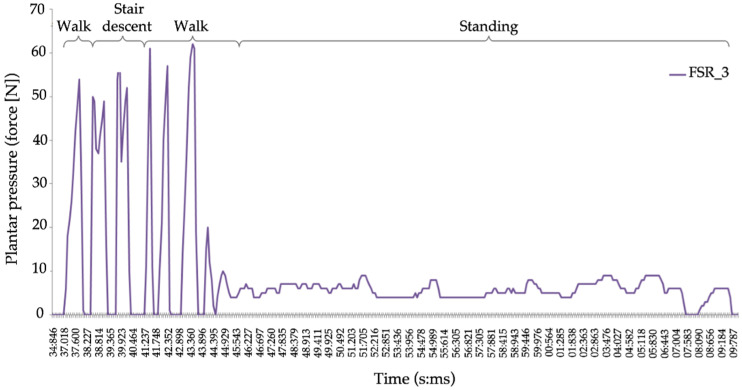
Sample dual-task plantar pressure was obtained from force sensitive resistor 3 (FSR_3) in the right shoe insole. This could be used to differentiate walking, stair descent and standing portions of the block. A similar procedure was used to extract the different blocks in the stair ascent conditions.

Data from the 12 force sensitive resistors (FSR) in each smart insole were collected at a frequency of 10 Hz. The SC condition was then used as a baseline to demonstrate the plantar pressure patterns applied on the FSRs when the participants were standing still compared to distinct patterns during overground walking, stair ascent, and stair descent [47] (Figure 3). Therefore, the average gait speed per condition could be derived from the distance the participant walked across the duration of the block.

### 2.6. Statistical Analyses

Mean hemodynamic responses in the PFC (∆HbO2 and ∆HbR) were examined using two 2 × 2 repeated measures ANOVAs to test for the main effects and interactions between task (SC or SM and DT) and stair ambulation condition (ascending or descending the stairs).

Gait speed was examined using a 2 × 2 repeated measures ANOVA to assess the mean group differences between task (SM and DT) and ambulation condition (up or down). In addition, the mean changes in response time and response accuracy during single and dual-tasks were measured using repeated measures ANOVAs across SC, DT_up_ and DT_down_.

A paired samples *t*-test was conducted to determine the mean differences in subjective emphasis placed on motor performance during dual-task ascent and descent tasks (DT_up_, DT_down_). Across all measures, gender was used as a between-subjects factor to assess differences in neural and performance measures between males and females [48]. Statistical significance was set at *p* < 0.05 and Bonferroni post-hoc tests were used to determine the location of significance for all ANOVAs. Means and standard deviations were also calculated for all participant characteristics and neuropsychological assessment scores to account for baseline cognitive functions.

## 3. Results

### 3.1. fNIRS Hemodynamic Response

Mean changes in cerebral oxygenation and deoxygenation did not significantly differ across channels or right and left PFC hemispheres (*p*-values > 0.070). Therefore, the ∆HbO2 and ∆HbR data were averaged across all channels to determine the changes in brain activity across the whole PFC. In addition, there were no significant differences between blocks of the same type (i.e., all the SC conditions) based on the order in which they were performed. As such, an average of each condition was calculated for the analyses. Across all measures, there were no significant gender differences.

**Figure 4:**
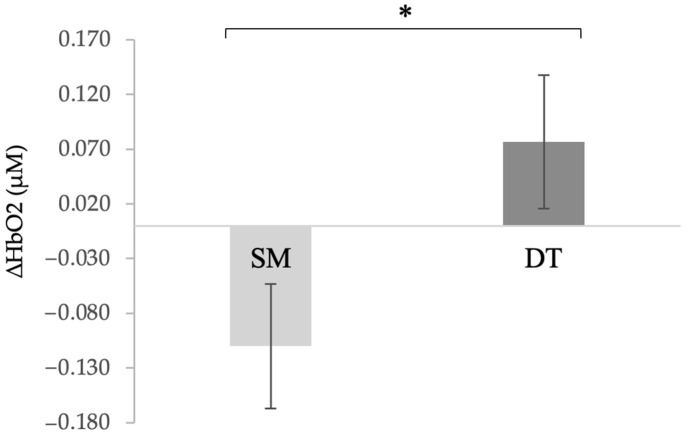
Mean prefrontal hemodynamic response change (∆HbO2) between single motor (SM) and dual-task (DT) blocks. ∆HbO2 significantly increased from SM to DT; *F* (1,19) = 6.46, *p* = 0.020, *η*^2^ = 0.254. (*) indicates significance *p* < 0.05 and error bars represent standard error of the mean.

∆HbO2: A repeated measures ANOVA revealed a main effect of tasks between SM and DT, *F* (1,19) = 6.46, *p* = 0.020, *η*^2^ = 0.254. Post-hoc analyses revealed that ∆HbO2 (µM) significantly increased (*p* = 0.020) from SM (*M* = −0.110 µM, *SD* = 0.057 µM) to DT (*M* = 0.077 µM, *SD* = 0.061 µM) (Figure 4). In addition, there were no significant effects of tasks between SC, DT_up_ and DT_down_ (*p* = 0.214).

∆HbR: A repeated measures ANOVA revealed that there were no significant main effects of SM task (*p* = 0.484), stair ascent (DT_up_) and descent (DT_down_) (*p* = 0.851) or interactions between SM, DT_up_ and DT_down_ (*p* = 0.549). Similarly, there were no significant differences between SC, DT_up_ and DT_down_ (*p* = 0.375) (Table 2).

### 3.2. Cognitive and Motor Performance

**Figure 5:**
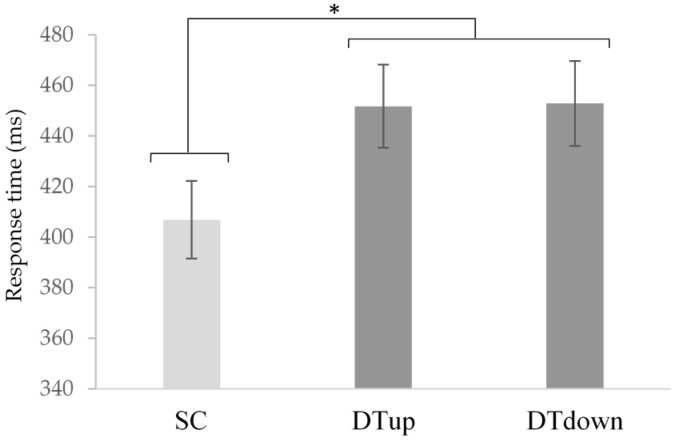
Mean vocal response times between single cognitive (SC) and dual-task (DT) up and down blocks. Response times were significantly slower during dual- compared to single tasks *F* (2,38) = 16.451, *p* < 0.001, *η*^2^ = 0.464. (*) indicates significance *p* < 0.001 and error bars represent standard error of the mean.

Vocal response time (ms): Results from a repeated measures ANOVA revealed a main effect of tasks for the SRT vocal response times, *F* (2,38) = 16.451, *p* < 0.001, *η*^2^ = 0.464 (Figure 5). Post-hoc analyses indicated that DT_up_ (*M* = 451.75 ms, *SD* = 73.69 ms, *p* < 0.001) and DT_down_ (*M* = 452.85 ms, *SD* = 75.07 ms) were significantly slower than SC (*M* = 406.85 ms, *SD* = 68.90 ms). However, DT_up_ and DT_down_ were not significantly different from one another (*p* = 1.00).

Accuracy (% correct): The repeated measures ANOVA revealed that there were no significant differences in accuracy between SC and DT_up_ and DT_down_ tasks (*p* = 0.135).

Gait speed (m/s): A repeated measures ANOVA indicated that there were no significant main effects between SM and DT (*p* = 0.356), ambulation condition (up/down) (*p* = 0.503) or interactions between task (SM/DT) and stair ambulation condition (up/down) (*p* = 0.475).

**Figure 6:**
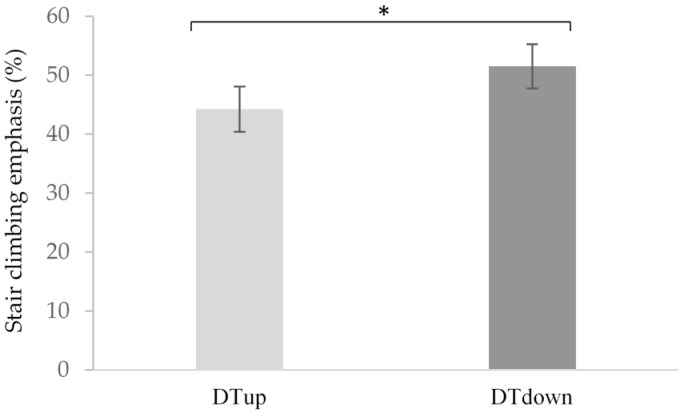
Participant responses to the subjective emphasis question. Participants reported focusing significantly more on stair climbing during stair descent (DT_down_) compared to ascent (DT_up_) *t* (19) = −2.214, *p* = 0.039. (*) indicates significance *p* < 0.05 and error bars represent standard error of the mean.

Dual-task motor emphasis (% emphasis): A *t*-test comparing subjective emphasis on stair ambulation during DT_up_ and DT_down_ revealed that significantly greater focus was placed on the motor task during dual-task stair descent (*M* = 51.5%, *SD* = 16.8%) compared to ascent (*M* = 44.3%, *SD* = 17.2%), *t* (19) = −2.214, *p* = 0.039 (Figure 6).

## 4. Discussion

The first aim of this study was to examine changes in prefrontal hemodynamic response during single and dual-task stair ascent and descent. The second aim was to measure the cognitive and motor performance costs using vocal response time, response accuracy and gait speed. After controlling for gender, findings revealed that cerebral oxygenation and vocal response time increased between single and dual- tasks, but there were no significant differences between cerebral deoxygenation, response accuracy and gait speed across single and dual-tasks or stair ascent and descent.

### 4.1. Hemodynamic Response: Single and Dual-Task

Brain activation during stair ambulation has been scarcely examined due to the limitations associated with stationary neuroimaging techniques. However, with advancements in portable neuroimaging such as fNIRS, many overground walking studies have identified that older adults exhibit increased brain activation during cognitive–motor dual-tasks [24]. Findings from this study support this; older adults exhibited bilateral PFC recruitment given that there were no hemodynamic response differences across the individual fNIRS channels or cerebral hemispheres. This is supported by STAC-r and neural compensation models, which suggest that older adults demonstrate widespread PFC activation to compensate for age-associated performance declines [31].

By examining the hemodynamic response across the whole PFC, there was a significant ∆HbO2 increase between the single and dual-tasks. This supports our initial hypothesis in that the competing demands of a cognitive and motor task required greater executive control compared to performing each one alone. Several studies examining cognitive performance have reported similar findings; pairing mental arithmetic [30,49], working memory [15,50], or verbal fluency tasks [45,46] with walking exercises resulted in greater prefrontal activation. In addition, studies examining obstacle negotiation, which may better reflect the challenges of stair ambulation, have also demonstrated increased PFC activation in older adults between single and dual-tasks [28,30,51].

A loss of automatic locomotor control with advancing age may equally contribute to greater PFC activation during stair ambulation [36,52]. A routine motor task such as walking without distractions may be automatically processed using minimal executive control [53]. These neural networks, found in the indirect locomotor pathway, are a quick and efficient way to process information during highly demanding situations [36]. However, a loss of automaticity may lead to greater cognitive–motor interference with controlled processes and may overwhelm the limited supply of processing resources. This may be the case for older adults during stair ascent and descent, which requires greater cognitive and motor control than usual walking. Thus, a loss of automaticity may lead to greater interference between stair ambulation and a secondary task and require greater recruitment of executive resources to produce the desired behaviour.

### 4.2. Hemodynamic Response: Ascent and Descent

Hemodynamic response changes during stair ambulation were expected to depend on the differing demands of stair ascent and descent compared to overground walking [1,17,28]. More specifically, stair descent was expected to recruit greater prefrontal resources because it is associated with greater motor planning and balance control than stair ascent [10,54]. Contrary to those expectations, findings from this study revealed that there were not any significant differences in PFC activation between stair ascent and descent. The primary reason for these findings may be the difficulty of the cognitive task. This study used a simple reaction time task that had participants respond to a random sequence of beeps. As demonstrated in the accuracy findings, the participants made few mistakes during both ascent and descent. This is supported by a study that determined that a working memory task did not diminish the participants’ capacity to dual-task [50]. As such, future studies should consider the degree of interference caused by a cognitive task to evaluate whether prefrontal activation changes between stair ascent and descent.

Additionally, the differences between ambulation conditions may have been too subtle to detect given the limited number of stairs used in this study. Other studies examining stair negotiation used three steps but did not measure brain activation [10,50,55]. An important difference that may facilitate stair ascent and descent is the amount of visual input acquired before reaching the first steps. Individuals can look up to four steps ahead during stair ascent and two steps ahead during stair descent [11]. However, because four steps were used in this study, the participants may have been able to plan for the entire staircase, thereby minimizing the challenges that differentiate stair ambulation conditions. Therefore, the number of stairs may play an indirect role in facilitating stair ambulation and diminish the differences between ascent and descent.

Another aspect that may have influenced the results is a fear of falling [9]. Previous research on this topic demonstrated increased prefrontal activation in older adults with a fear of falling [46,56]. To account for this, participants completed the FES-I questionnaire in which they rated their overall concerns with falling on a scale from “not concerned at all” to “very concerned”. The older adults reported a low fear of falling, and more specifically, the majority selected “not concerned” or “not concerned at all” to describe their experience on stairs. This may account for the insignificant differences between stair ascent and descent; these particular older adults were not concerned with falling on stairs. In addition, certain safety measures such as the harness worn by participants may have minimized the participants’ fear of falling [57]. In contrast, the effects of using a handrail to facilitate stair ascent and descent are mixed. For instance, handrails may only increase stability during stair descent in older adults who report a fear of falling [58]. Others suggest simply touching a handrail and light handrail usage provide an external frame of reference to maintain body orientation and balance [12,59]. Therefore, having these external aids may have minimized the detectable differences between stair ascent and descent.

### 4.3. Cognitive Performance: Vocal Response Time and Accuracy

Cognitive performance may be used to measure behavioural outcomes that accompany neural activation. Dual-task costs, or a decline in performance between single and dual-tasks, are expected when two interfering tasks compete for the same cognitive resources [2,16]. This study measured vocal response time during a simple reaction time task. Findings revealed that single task vocal response time was significantly faster than dual-task ascent and descent, but the dual-tasks were not significantly different from one another. Furthermore, accuracy was measured based on the number of correct responses provided by the participants out of the total possible correct responses. The accuracy findings demonstrated that there were no significant differences in response accuracy between single and dual-tasks or between ascent and descent.

Firstly, vocal response time was expected to decrease between the single and dual-tasks due to increased cognitive–motor interference associated with dual-tasking. This has been demonstrated in a standing balance task, where older adults exhibited slower response times with increasing postural task complexity [18]. In this same study, response time increased with a decreased base of support. These findings may be extended to stair ambulation—ascending and descending stairs may cause greater instability due to a decreased base of support compared to standing or overground walking. Taken together with the neural findings, increased PFC activation may demonstrate inefficient compensation given that response time increased during the dual-task. Therefore, the increased recruitment of prefrontal resources did not support cognitive performance maintenance between single and dual-tasks.

Similar to the neural findings, there were not any significant differences in vocal response time between stair ascent and descent. A similar study that demonstrated similar vocal response times during ascent and descent attributed their findings to their participant group, which was highly fit and reported a low fear of falling [17]. Following these criteria, our sample may equally be considered higher functioning, in which differences between stair ascent and descent may be less prominent. Our sample was also composed of highly educated older adults with no cognitive impairment. Therefore, the differences between stair ascent and descent may be more prominent in frail older adults or those who have a fear of falling.

In comparison, response accuracy remained very high despite the decrease in response time. As mentioned previously, the cognitive task was very simple and likely did not challenge the participants enough to elicit accuracy differences. There were no significant speed–accuracy trade-offs between response time and response accuracy. However, longer response times allow for a greater amount of time to process the stimuli to ensure a correct response is given. Older adults tend to employ this strategy, which places greater emphasis on responding correctly rather than responding quickly [60]. This may account for accuracy performance maintenance while response times increased during the dual-tasks. Similarly, this process is mediated by the PFC, and may have contributed to increased interference and thus brain activation in the dual- versus single task [61].

### 4.4. Motor Performance: Gait Speed

Numerous studies have reported on the relationship between decreased executive functions and worse gait performance in older adults [5,6,52]. In line with these studies, gait speed was expected to decrease between single and dual-tasks due to the competing demands of cognitive and motor tasks. Furthermore, slower gait speed was expected during stair ascent versus descent due to the physical challenges associated with ascent [12,13]. However, findings from this study revealed that there were not any significant differences in gait speed between tasks and stair ambulation conditions. In other words, the older adults were able to maintain their motor performance despite the increase in interference and task complexity.

When faced with simultaneous cognitive and motor tasks, older adults tend to prioritize motor performance [62]. Moreover, when given the choice between a cognitive or motor performance aid, older adults select the option to supplement gait performance [63]. This is known as a “posture-first” strategy, in which older adults minimize decrements in gait performance by prioritizing posture over cognitive task performance [62,63]. Gait speed findings from our study revealed that the older adults exhibited a posture-first strategy, and gait speed was maintained throughout the single and dual-tasks. Interestingly, the participants subjectively reported placing more emphasis on motor performance during dual-task descent than ascent. Perhaps this was necessary given the challenges associated with stair descent, and that individuals who demonstrate a posture-second strategy face greater challenges with obstacle avoidance and may be more prone to falling [51]. Therefore, compared to the neural and response time findings, a posture-first strategy may protect older adults from a breakdown of cognitive and motor performance during a complex task. In this study, gait speed maintenance across tasks emphasizes the importance of examining neural and performance measures simultaneously, in that neural activation changes may precede observable differences in motor performance.

Furthermore, the insignificant gait speed differences between ascent and descent may be explained by certain features of this study. The physical demands of stair ascent were expected to promote slower gait speed; however, the number of stairs used in this study and the highly functioning participant group may have minimized the expected difficulty effects. A study that investigated gait speed changes across multiple flights of stairs determined that gait speed only declined following many stairs once participants were fatigued [64]. The present study only employed four stairs and participants were offered breaks to ensure that they were not fatigued. Similarly, the sample consisted of healthy older adults, which may account for their ability to maintain a high level of motor performance. Gait speed findings across studies using fewer than four stairs remain inconsistent [55]. For example, one study demonstrated that stair ascent and descent duration were significantly slower in older adults when stair negotiation was paired with a difficult working memory task [50]. However, there were no significant differences between stair ascent and descent alone compared to dual-tasking with an easy working memory task. Another study revealed that responding to an arithmetic task led to a decline in gait speed between single and dual-tasks during stair descent [20]. Therefore, gait speed changes may greatly depend on the difficulty of the cognitive task. In the present study, the cognitive task may have insufficiently challenged the older adults to cause a substantial difference in motor performance between stair ascent and descent.

### 4.5. Limitations

The first limitation of this study is the participant group which was made up of healthy older adults who reported a low fear of falling. This may limit the generalizability of these findings to larger groups of older adults. Older adults who demonstrate difficulties with stair ambulation may have underlying health conditions or trouble with balance and cognition. Therefore, a more diverse group of older adults should be examined to better understand the neural and behavioural changes during stair ascent and descent in older adults.

Lastly, this study specifically examined PFC activity. However, other cortical and subcortical regions involved in motor control or visual processing (e.g., premotor cortex [65], sensorimotor cortex [66] and supplementary motor area [67]) should be further examined to understand their role in stair ambulation in older adults. This may reveal more subtle differences across stair ambulation conditions and how cognitive resources are shared amongst different brain regions.

## 5. Conclusions

Ambulating stairs is an important part of everyday life but remains a safety concern for older adults. This may be due to decreased executive functioning and less efficient cognitive and motor processes. Few studies have simultaneously examined brain activation and performance outcomes during stair ambulation due to the limitations associated with mobile neuroimaging techniques. fNIRS, however, revealed that older adults displayed increased PFC activation and slower response times between single and dual-tasks. Conversely, accuracy and gait speed performance remained unchanged despite increased PFC activation. These changes highlight the importance of examining neural and performance measures simultaneously to monitor neural changes that may precede observable declines in performance with age. However, in both neural and behavioural measures, stair ascent and descent were not significantly different. This may be due to the difficulty of the cognitive task, the number of stairs, or the participants’ minimal concern with falling. Future studies should consider these factors in their study design to better understand the neural correlates of dual-task stair climbing in older adults.

## Figures and Tables

**Table 1:** Characteristics of study participants and neuropsychological test scores.

Variable	Sample (*n* = 20) Mean (*SD*)
Age (years)	72.7 (6.9)
Gender (F:M)	14:6
Education (years)	17.3 (2.4)
GDS (/30)	3.3 (3.2)
FES-I (/64)	23.4 (7.6)
Fear going up or down stairs ^1^ (/4)	1.7 (0.7)
MoCA (/30)	27.3 (1.4)
Digit forward (/16)	10.4 (1.6)
Digit backward (/14)	7.4 (1.7)
Digit Symbol (/93)	44.5 (13.4)
TMT A (s)	39.3 (14.8)
TMT B (s)	82.3 (34.9)

GDS: Geriatric Depression Scale; FES-I: Falls Efficacy Scale—International; MoCA: Montreal Cognitive Assessment; TMT: Trail Making Test. ^1^ Question extracted from FES-I.

**Table 2:** Mean and standard deviation values, *M* (*SD*) for neural, cognitive and performance measures across single and dual-task blocks.

Variable	SC	SM_up_	SM_down_	DT_up_	DT_down_
∆HbO2 (µM)	−0.04 (0.23)	−0.01 (0.31)	−0.21 (0.33)	0.11 (0.35)	0.04 (0.31)
∆HbR (µM)	−0.02 (0.08)	−0.02 (0.12)	0.01 (0.12)	0.02 (0.10)	0.01 (0.13)
Response time (ms)	406.9 (68.9)	.	.	451.8 (73.7)	452.9 (75.1)
Accuracy (%)	100.0 (0.0)	.	.	99.3 (3.1)	98.6 (3.7)
Gait speed (m/s)	.	0.62 (0.12)	0.63 (0.12)	0.60 (0.12)	0.62 (0.13)

## Data Availability

Data available on request due to privacy and ethical restrictions.
